# 1-[(2-Hy­droxy­phen­yl)(pyrrolidin-1-yl)­meth­yl]naphthalen-2-ol *N*,*N*-dimethyl­formamide monosolvate

**DOI:** 10.1107/S1600536812007787

**Published:** 2012-02-29

**Authors:** Wen-xiang Wang

**Affiliations:** a Ordered Matter Science Research Center, College of Chemistry and Chemical Engineering, Southeast University, Nanjing 211189, People’s Republic of China

## Abstract

The title compound, C_21_H_21_NO_2_·C_3_H_7_NO, was synthesized by solvent-free one-pot three-component reaction of naphthalen-2-ol, 2-hy­droxy­benzaldehyde and pyrrolidine. The dihedral angle between the naphthalene ring system and the benzene ring is 77.74 (6)°. The pyrrolidine ring assumes an envelope conformation. An intra­molecular O—H⋯N and an inter­molecular O—H⋯O hydrogen bond are observed.

## Related literature

For background to Betti-type reactions, see: Pu & Yu (2001 ▶); Yuan (2005 ▶). For ring puckering parameters, see: Cremer & Pople (1975 ▶).
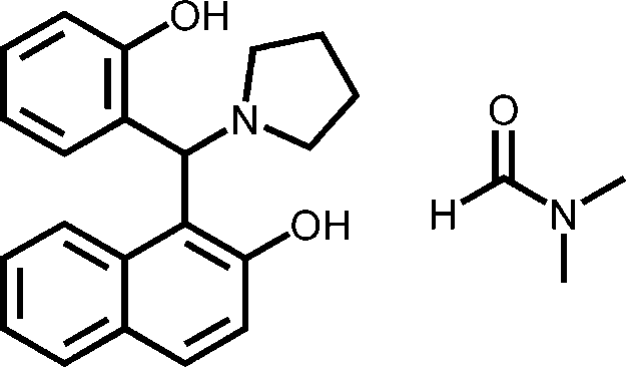


## Experimental

### 

#### Crystal data

C_21_H_21_NO_2_·C_3_H_7_NO
*M* *_r_* = 392.48
Monoclinic, 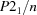

*a* = 13.675 (3) Å
*b* = 9.6518 (19) Å
*c* = 16.505 (3) Å
β = 102.84 (3)°
*V* = 2123.9 (7) Å^3^
*Z* = 4
Mo *K*α radiation
μ = 0.08 mm^−1^
*T* = 293 K
0.25 × 0.22 × 0.20 mm

#### Data collection

Rigaku Mercury2 (2 × 2 bin mode) diffractometer
Absorption correction: multi-scan (*CrystalClear*; Rigaku, 2005 ▶) *T*_min_ = 0.963, *T*_max_ = 0.989
21445 measured reflections
4874 independent reflections
2223 reflections with *I* > 2σ(*I*)
*R*_int_ = 0.100

#### Refinement

*R*[*F* ^2^ > 2σ(*F* ^2^)] = 0.066
*wR*(*F* ^2^) = 0.184
*S* = 1.03
4874 reflections
262 parameters
H-atom parameters constrained
Δρ_max_ = 0.20 e Å^−3^
Δρ_min_ = −0.17 e Å^−3^

### 

Data collection: *CrystalClear* (Rigaku, 2005 ▶); cell refinement: *CrystalClear*; data reduction: *CrystalClear*; program(s) used to solve structure: *SHELXS97* (Sheldrick, 2008 ▶); program(s) used to refine structure: *SHELXL97* (Sheldrick, 2008 ▶); molecular graphics: *SHELXTL* (Sheldrick, 2008 ▶); software used to prepare material for publication: *SHELXL97*.

## Supplementary Material

Crystal structure: contains datablock(s) I, global. DOI: 10.1107/S1600536812007787/rz2712sup1.cif

Structure factors: contains datablock(s) I. DOI: 10.1107/S1600536812007787/rz2712Isup2.hkl

Supplementary material file. DOI: 10.1107/S1600536812007787/rz2712Isup3.cml

Additional supplementary materials:  crystallographic information; 3D view; checkCIF report

## Figures and Tables

**Table 1:** Hydrogen-bond geometry (Å, °)

*D*—H⋯*A*	*D*—H	H⋯*A*	*D*⋯*A*	*D*—H⋯*A*
O2—H2*A*⋯N1	0.82	1.88	2.565 (3)	140
O3—H3*A*⋯O1	0.82	1.86	2.678 (3)	173

**Fig. 1.**
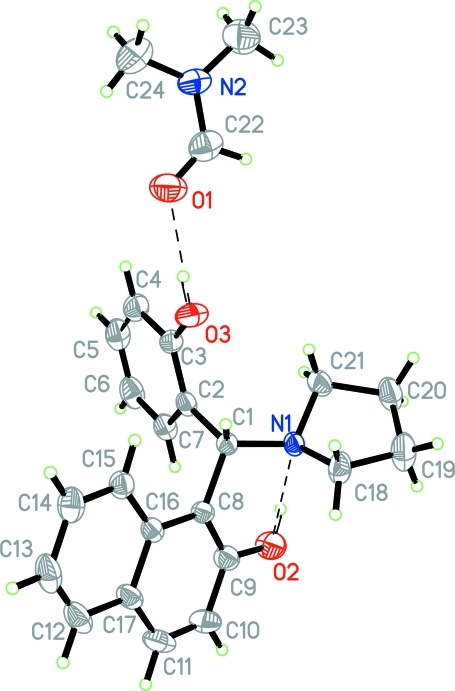
The molecular structure of the title compound with displacement ellipsoids drawn at the 30% probability level. Intra- and intermolecular hydrogen bonds are shown as dashed lines.

## supplementary crystallographic information

### Comment

The so-called Betti base derivatives, which can be synthesized by many ways (Pu & Yu, 2001; Yuan, 2005), have been of great interest in coordination chemistry. Herein the crystal structure of one such compounds obtained by solvent free, one-pot, three-component domino reaction of naphthalen-2-ol, 2-hydroxybenzaldehyde and pyrrolidine is reported.

In the molecule of the title compound (Fig. 1) the bond lengths and angles are well within the expected range. The dihedral angle between the naphthalene ring system and the benzene ring is 77.74 (6)°. The pyrrolidine ring adopts an envelope conformation, with puckering parameters (Cremer & Pople, 1975) *Q* = 0.408 (3) Å and φ = 170.7 (5)°. An intramolecular O—H···N hydrogen bond (Table 1) stabilizes the molecular conformation. In the crystal structure, the molecules interact *via* an intermolecular O—H···O hydrogen bond (Table 1).

### Experimental

A dry 50 ml flask was charged with 2-hydroxybenzaldehyde (10 mmol), naphthalen-2-ol (10 mmol) and pyrrolidine (10 mmol). The mixture was stirred at 100°C for 5 h, then ethanol (15 ml) was added. After refluxing for 30 minutes, the precipitate was filtrated out, washed with ethanol for 3 times and purified by recrystallization from a mixed solution of dichloromethane, methanol and DMF (30:8:10 *v*/*v*/*v*) to give crystals of the title compound suitable for X-ray analysis.

### Refinement

All H atoms were calculated geometrically and refined using a riding model, with C—H = 0.93–0.98 Å, O—H = 0.82 Å, and with *U*_iso_(H) = 1.2 *U*_eq_(C) or 1.5 *U*_eq_(C, O) for methyl and hydroxy H atoms.

### Crystal data

**Table:**

C_21_H_21_NO_2_·C_3_H_7_NO	*F*(000) = 840
*M**_r_* = 392.48	*D*_x_ = 1.227 Mg m^−^^3^
Monoclinic, *P*2_1_/*n*	Mo *K*α radiation, λ = 0.71073 Å
Hall symbol: -P 2yn	Cell parameters from 3860 reflections
*a* = 13.675 (3) Å	θ = 2.3–27.5°
*b* = 9.6518 (19) Å	µ = 0.08 mm^−^^1^
*c* = 16.505 (3) Å	*T* = 293 K
β = 102.84 (3)°	Prism, colourless
*V* = 2123.9 (7) Å^3^	0.25 × 0.22 × 0.20 mm
*Z* = 4	

### Data collection

**Table:**

Rigaku Mercury2 (2x2 bin mode) diffractometer	4874 independent reflections
Radiation source: fine-focus sealed tube	2223 reflections with *I* > 2σ(*I*)
Graphite monochromator	*R*_int_ = 0.100
Detector resolution: 13.6612 pixels mm^-1^	θ_max_ = 27.5°, θ_min_ = 3.0°
CCD Profile fitting scans	*h* = −17→17
Absorption correction: multi-scan (*CrystalClear*; Rigaku, 2005)	*k* = −12→12
*T*_min_ = 0.963, *T*_max_ = 0.989	*l* = −21→21
21445 measured reflections	

### Refinement

**Table:**

Refinement on *F*^2^	Primary atom site location: structure-invariant direct methods
Least-squares matrix: full	Secondary atom site location: difference Fourier map
*R*[*F*^2^ > 2σ(*F*^2^)] = 0.066	Hydrogen site location: inferred from neighbouring sites
*wR*(*F*^2^) = 0.184	H-atom parameters constrained
*S* = 1.03	*w* = 1/[σ^2^(*F*_o_^2^) + (0.0731*P*)^2^ + 0.0577*P*] where *P* = (*F*_o_^2^ + 2*F*_c_^2^)/3
4874 reflections	(Δ/σ)_max_ < 0.001
262 parameters	Δρ_max_ = 0.20 e Å^−^^3^
0 restraints	Δρ_min_ = −0.17 e Å^−^^3^

### Special details

**Table:**

Geometry. All e.s.d.'s (except the e.s.d. in the dihedral angle between two l.s. planes) are estimated using the full covariance matrix. The cell e.s.d.'s are taken into account individually in the estimation of e.s.d.'s in distances, angles and torsion angles; correlations between e.s.d.'s in cell parameters are only used when they are defined by crystal symmetry. An approximate (isotropic) treatment of cell e.s.d.'s is used for estimating e.s.d.'s involving l.s. planes.
Refinement. Refinement of *F*^2^ against ALL reflections. The weighted *R*-factor *wR* and goodness of fit *S* are based on *F*^2^, conventional *R*-factors *R* are based on *F*, with *F* set to zero for negative *F*^2^. The threshold expression of *F*^2^ > σ(*F*^2^) is used only for calculating *R*-factors(gt) *etc*. and is not relevant to the choice of reflections for refinement. *R*-factors based on *F*^2^ are statistically about twice as large as those based on *F*, and *R*- factors based on ALL data will be even larger.

### Fractional atomic coordinates and isotropic or equivalent isotropic displacement parameters (Å^2^)

**Table:**

	*x*	*y*	*z*	*U*_iso_*/*U*_eq_	
O2	0.86622 (14)	0.7318 (2)	1.01567 (12)	0.0696 (6)	
H2A	0.8484	0.7762	1.0522	0.104*	
O3	0.59379 (14)	1.15741 (19)	1.04615 (12)	0.0620 (6)	
H3A	0.5723	1.2281	1.0636	0.093*	
N1	0.73008 (15)	0.8368 (2)	1.08164 (11)	0.0421 (5)	
C8	0.72196 (18)	0.8476 (3)	0.93131 (15)	0.0396 (6)	
C16	0.66100 (19)	0.8697 (2)	0.84998 (15)	0.0414 (6)	
C15	0.5776 (2)	0.9595 (3)	0.83292 (16)	0.0508 (7)	
H15A	0.5614	1.0099	0.8761	0.061*	
C1	0.69749 (19)	0.9227 (2)	1.00552 (14)	0.0397 (6)	
H1A	0.6245	0.9335	0.9952	0.048*	
C9	0.8026 (2)	0.7581 (3)	0.94091 (17)	0.0508 (7)	
C17	0.6846 (2)	0.7971 (3)	0.78094 (16)	0.0518 (7)	
C7	0.8420 (2)	1.0914 (3)	1.00816 (15)	0.0540 (7)	
H7A	0.8790	1.0181	0.9940	0.065*	
C2	0.7445 (2)	1.0671 (3)	1.01694 (14)	0.0422 (6)	
C3	0.6901 (2)	1.1791 (3)	1.03819 (15)	0.0485 (7)	
C10	0.8248 (2)	0.6866 (3)	0.8726 (2)	0.0648 (9)	
H10A	0.8794	0.6267	0.8806	0.078*	
C14	0.5201 (2)	0.9739 (3)	0.75428 (19)	0.0639 (8)	
H14A	0.4650	1.0330	0.7451	0.077*	
C5	0.8301 (3)	1.3300 (3)	1.04007 (18)	0.0706 (10)	
H5A	0.8585	1.4179	1.0477	0.085*	
C12	0.6236 (3)	0.8158 (4)	0.70070 (17)	0.0679 (9)	
H12A	0.6392	0.7685	0.6561	0.082*	
C13	0.5429 (3)	0.9011 (4)	0.68713 (19)	0.0734 (10)	
H13A	0.5030	0.9113	0.6340	0.088*	
C18	0.6664 (2)	0.7135 (3)	1.08117 (17)	0.0628 (9)	
H18A	0.6778	0.6458	1.0409	0.075*	
H18B	0.5958	0.7382	1.0690	0.075*	
C11	0.7670 (2)	0.7048 (3)	0.7955 (2)	0.0651 (9)	
H11A	0.7817	0.6557	0.7512	0.078*	
C21	0.7284 (2)	0.9051 (3)	1.16070 (15)	0.0596 (8)	
H21A	0.6674	0.9587	1.1564	0.072*	
H21B	0.7858	0.9658	1.1778	0.072*	
C4	0.7332 (3)	1.3106 (3)	1.04918 (17)	0.0636 (9)	
H4A	0.6967	1.3851	1.0626	0.076*	
C20	0.7325 (3)	0.7847 (3)	1.22155 (18)	0.0734 (10)	
H20A	0.8000	0.7729	1.2547	0.088*	
H20B	0.6876	0.8013	1.2585	0.088*	
C19	0.7004 (3)	0.6593 (4)	1.16925 (18)	0.0850 (11)	
H19A	0.6458	0.6123	1.1867	0.102*	
H19B	0.7558	0.5951	1.1732	0.102*	
C6	0.8852 (2)	1.2218 (4)	1.01990 (18)	0.0667 (9)	
H6A	0.9507	1.2357	1.0142	0.080*	
N2	0.45935 (18)	1.5046 (3)	1.20142 (17)	0.0669 (7)	
O1	0.50827 (19)	1.3762 (2)	1.10246 (16)	0.0883 (8)	
C22	0.5038 (2)	1.3964 (4)	1.1737 (3)	0.0729 (9)	
H22A	0.5340	1.3312	1.2128	0.087*	
C23	0.4627 (3)	1.5224 (4)	1.2890 (2)	0.1042 (13)	
H23A	0.5010	1.4487	1.3198	0.156*	
H23C	0.3957	1.5206	1.2979	0.156*	
H23D	0.4934	1.6096	1.3073	0.156*	
C24	0.4053 (3)	1.6046 (4)	1.1449 (3)	0.1163 (15)	
H24C	0.4091	1.5807	1.0892	0.174*	
H24D	0.4342	1.6946	1.1585	0.174*	
H24A	0.3364	1.6056	1.1491	0.174*	

### Atomic displacement parameters (Å^2^)

**Table:**

	*U*^11^	*U*^22^	*U*^33^	*U*^12^	*U*^13^	*U*^23^
O2	0.0655 (13)	0.0786 (15)	0.0595 (14)	0.0240 (11)	0.0028 (11)	−0.0003 (11)
O3	0.0592 (13)	0.0468 (12)	0.0864 (15)	−0.0006 (10)	0.0300 (11)	−0.0142 (10)
N1	0.0557 (13)	0.0384 (12)	0.0315 (11)	−0.0083 (11)	0.0085 (10)	0.0001 (9)
C8	0.0430 (15)	0.0391 (15)	0.0381 (15)	−0.0027 (12)	0.0116 (12)	−0.0032 (11)
C16	0.0480 (16)	0.0391 (15)	0.0385 (15)	−0.0134 (13)	0.0125 (12)	−0.0023 (11)
C15	0.0569 (18)	0.0499 (18)	0.0439 (16)	−0.0025 (14)	0.0077 (14)	0.0028 (13)
C1	0.0474 (15)	0.0372 (15)	0.0346 (14)	−0.0019 (12)	0.0090 (11)	−0.0002 (11)
C9	0.0529 (17)	0.0527 (18)	0.0465 (17)	0.0006 (15)	0.0105 (14)	−0.0028 (14)
C17	0.0662 (19)	0.0539 (18)	0.0368 (16)	−0.0154 (15)	0.0146 (14)	−0.0106 (13)
C7	0.0608 (19)	0.0584 (19)	0.0433 (16)	−0.0102 (15)	0.0130 (14)	0.0009 (14)
C2	0.0505 (16)	0.0446 (17)	0.0309 (14)	−0.0086 (13)	0.0079 (12)	0.0009 (11)
C3	0.0684 (19)	0.0371 (16)	0.0394 (15)	−0.0053 (14)	0.0108 (14)	−0.0048 (12)
C10	0.064 (2)	0.067 (2)	0.069 (2)	0.0125 (16)	0.0265 (18)	−0.0124 (17)
C14	0.0599 (19)	0.069 (2)	0.056 (2)	−0.0037 (16)	−0.0010 (16)	0.0133 (16)
C5	0.102 (3)	0.054 (2)	0.0509 (19)	−0.035 (2)	0.0074 (19)	−0.0035 (15)
C12	0.093 (3)	0.075 (2)	0.0352 (18)	−0.031 (2)	0.0135 (17)	−0.0097 (15)
C13	0.087 (3)	0.084 (3)	0.0406 (19)	−0.026 (2)	−0.0045 (17)	0.0112 (17)
C18	0.084 (2)	0.0501 (18)	0.0505 (18)	−0.0236 (16)	0.0062 (15)	0.0073 (14)
C11	0.075 (2)	0.066 (2)	0.064 (2)	−0.0113 (18)	0.0362 (18)	−0.0245 (16)
C21	0.085 (2)	0.0585 (19)	0.0348 (15)	−0.0073 (17)	0.0131 (15)	−0.0049 (14)
C4	0.093 (3)	0.0411 (18)	0.0552 (19)	−0.0131 (17)	0.0138 (17)	−0.0047 (14)
C20	0.094 (2)	0.080 (2)	0.0452 (18)	−0.0161 (19)	0.0134 (17)	0.0097 (17)
C19	0.114 (3)	0.077 (2)	0.058 (2)	−0.030 (2)	0.006 (2)	0.0151 (19)
C6	0.071 (2)	0.077 (2)	0.0511 (19)	−0.030 (2)	0.0121 (16)	0.0032 (17)
N2	0.0663 (17)	0.0551 (17)	0.082 (2)	0.0039 (14)	0.0228 (15)	−0.0131 (15)
O1	0.1021 (19)	0.0734 (17)	0.101 (2)	−0.0031 (13)	0.0463 (16)	−0.0201 (15)
C22	0.065 (2)	0.056 (2)	0.097 (3)	−0.0124 (17)	0.018 (2)	−0.010 (2)
C23	0.136 (4)	0.094 (3)	0.084 (3)	−0.007 (3)	0.028 (2)	−0.020 (2)
C24	0.130 (4)	0.099 (3)	0.119 (3)	0.048 (3)	0.027 (3)	0.023 (3)

### Geometric parameters (Å, º)

**Table:**

O2—C9	1.366 (3)	C5—H5A	0.9300
O2—H2A	0.8200	C12—C13	1.356 (4)
O3—C3	1.368 (3)	C12—H12A	0.9300
O3—H3A	0.8200	C13—H13A	0.9300
N1—C21	1.467 (3)	C18—C19	1.518 (4)
N1—C18	1.473 (3)	C18—H18A	0.9700
N1—C1	1.489 (3)	C18—H18B	0.9700
C8—C9	1.382 (3)	C11—H11A	0.9300
C8—C16	1.430 (3)	C21—C20	1.529 (4)
C8—C1	1.523 (3)	C21—H21A	0.9700
C16—C15	1.410 (4)	C21—H21B	0.9700
C16—C17	1.434 (3)	C4—H4A	0.9300
C15—C14	1.367 (4)	C20—C19	1.495 (4)
C15—H15A	0.9300	C20—H20A	0.9700
C1—C2	1.529 (3)	C20—H20B	0.9700
C1—H1A	0.9800	C19—H19A	0.9700
C9—C10	1.411 (4)	C19—H19B	0.9700
C17—C12	1.411 (4)	C6—H6A	0.9300
C17—C11	1.415 (4)	N2—C22	1.339 (4)
C7—C6	1.386 (4)	N2—C24	1.428 (4)
C7—C2	1.393 (4)	N2—C23	1.446 (4)
C7—H7A	0.9300	O1—C22	1.207 (4)
C2—C3	1.400 (4)	C22—H22A	0.9300
C3—C4	1.394 (4)	C23—H23A	0.9600
C10—C11	1.351 (4)	C23—H23C	0.9600
C10—H10A	0.9300	C23—H23D	0.9600
C14—C13	1.404 (4)	C24—H24C	0.9600
C14—H14A	0.9300	C24—H24D	0.9600
C5—C6	1.372 (4)	C24—H24A	0.9600
C5—C4	1.379 (4)		
			
C9—O2—H2A	109.5	N1—C18—H18A	111.2
C3—O3—H3A	109.5	C19—C18—H18A	111.2
C21—N1—C18	104.0 (2)	N1—C18—H18B	111.2
C21—N1—C1	116.0 (2)	C19—C18—H18B	111.2
C18—N1—C1	112.29 (19)	H18A—C18—H18B	109.2
C9—C8—C16	119.0 (2)	C10—C11—C17	121.3 (3)
C9—C8—C1	121.2 (2)	C10—C11—H11A	119.4
C16—C8—C1	119.8 (2)	C17—C11—H11A	119.4
C15—C16—C8	123.8 (2)	N1—C21—C20	103.7 (2)
C15—C16—C17	117.1 (2)	N1—C21—H21A	111.0
C8—C16—C17	119.1 (2)	C20—C21—H21A	111.0
C14—C15—C16	121.4 (3)	N1—C21—H21B	111.0
C14—C15—H15A	119.3	C20—C21—H21B	111.0
C16—C15—H15A	119.3	H21A—C21—H21B	109.0
N1—C1—C8	109.61 (19)	C5—C4—C3	119.9 (3)
N1—C1—C2	111.41 (19)	C5—C4—H4A	120.0
C8—C1—C2	111.8 (2)	C3—C4—H4A	120.0
N1—C1—H1A	107.9	C19—C20—C21	105.9 (2)
C8—C1—H1A	107.9	C19—C20—H20A	110.6
C2—C1—H1A	107.9	C21—C20—H20A	110.6
O2—C9—C8	123.5 (2)	C19—C20—H20B	110.6
O2—C9—C10	115.1 (3)	C21—C20—H20B	110.6
C8—C9—C10	121.5 (3)	H20A—C20—H20B	108.7
C12—C17—C11	121.5 (3)	C20—C19—C18	105.2 (3)
C12—C17—C16	119.6 (3)	C20—C19—H19A	110.7
C11—C17—C16	118.9 (3)	C18—C19—H19A	110.7
C6—C7—C2	121.8 (3)	C20—C19—H19B	110.7
C6—C7—H7A	119.1	C18—C19—H19B	110.7
C2—C7—H7A	119.1	H19A—C19—H19B	108.8
C7—C2—C3	118.1 (2)	C5—C6—C7	119.0 (3)
C7—C2—C1	121.7 (2)	C5—C6—H6A	120.5
C3—C2—C1	120.2 (2)	C7—C6—H6A	120.5
O3—C3—C4	121.0 (3)	C22—N2—C24	120.8 (3)
O3—C3—C2	118.8 (2)	C22—N2—C23	121.2 (3)
C4—C3—C2	120.1 (3)	C24—N2—C23	118.0 (3)
C11—C10—C9	120.3 (3)	O1—C22—N2	125.8 (4)
C11—C10—H10A	119.9	O1—C22—H22A	117.1
C9—C10—H10A	119.9	N2—C22—H22A	117.1
C15—C14—C13	121.1 (3)	N2—C23—H23A	109.5
C15—C14—H14A	119.4	N2—C23—H23C	109.5
C13—C14—H14A	119.4	H23A—C23—H23C	109.5
C6—C5—C4	121.1 (3)	N2—C23—H23D	109.5
C6—C5—H5A	119.5	H23A—C23—H23D	109.5
C4—C5—H5A	119.5	H23C—C23—H23D	109.5
C13—C12—C17	121.5 (3)	N2—C24—H24C	109.5
C13—C12—H12A	119.3	N2—C24—H24D	109.5
C17—C12—H12A	119.3	H24C—C24—H24D	109.5
C12—C13—C14	119.3 (3)	N2—C24—H24A	109.5
C12—C13—H13A	120.4	H24C—C24—H24A	109.5
C14—C13—H13A	120.4	H24D—C24—H24A	109.5
N1—C18—C19	102.6 (2)		

### Hydrogen-bond geometry (Å, º)

**Table:**

*D*—H···*A*	*D*—H	H···*A*	*D*···*A*	*D*—H···*A*
O2—H2*A*···N1	0.82	1.88	2.565 (3)	140
O3—H3*A*···O1	0.82	1.86	2.678 (3)	173
